# Retraction: Cytokinin and abiotic stress tolerance -what has been accomplished and the way forward?

**DOI:** 10.3389/fgene.2024.1399259

**Published:** 2024-03-19

**Authors:** Frontiers Editorial Office

Following publication, the publisher found evidence of peer review manipulation. As the scientific integrity of the article cannot be guaranteed, and in adherence to the recommendations of the Committee on Publication Ethics (COPE), the article is retracted.

This retraction was approved by the Chief Executive Editor of Frontiers. The authors received a communication regarding the retraction and had a chance to respond. This communication has been recorded by the publisher.

Frontiers would like to thank the concerned reader who contacted us regarding the published article and the users on PubPeer for bringing the published article to our attention.

